# Nachruf auf Prof. Dr. Martin Schumacher (1944–2021)

**DOI:** 10.1007/s00062-021-01135-w

**Published:** 2022-01-18

**Authors:** Ansgar Berlis

**Affiliations:** Neuroradiologie, Universitätsklinikum Augsburg, Augsburg, Deutschland


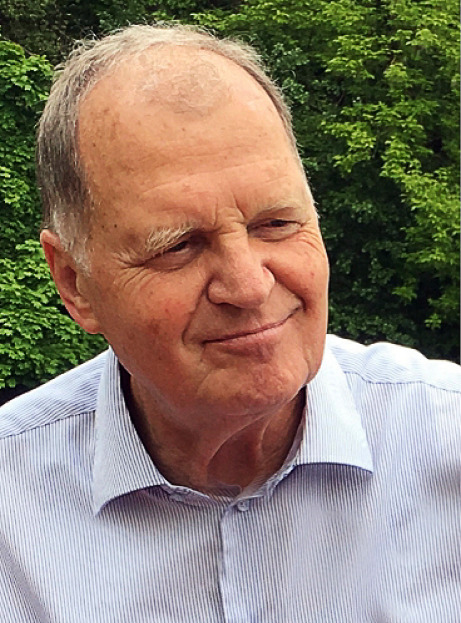


Am 18.11.2021 starb Professor emeritus Dr. med. Martin Schumacher, ehemaliger Leiter der Neuroradiologie des Universitätsklinikums Freiburg, im Alter von 77 Jahren.

Martin Schumacher studierte von 1965 bis 1971 Medizin in Freiburg und promovierte 1971 nach einem Forschungsaufenthalt 1970 in den USA zum Dr. med. Es folgten die Medizinalassistentenzeit und die Weiterbildung zum Facharzt für Neurologie an der Neurologischen Universitätsklinik Freiburg i. Br. bei Prof. Jung. Ergänzt wurde die Ausbildung durch seine Tätigkeit in der Neurochirurgischen Universitätsklinik Freiburg bei Prof. Seeger. 1977 wechselte Prof. Schumacher nach Tübingen und habilitierte 1980 über das Thema „Neuroradiologie experimenteller Hirntumore“. 1981 wurde er Chefarzt der Abteilung Neuroradiologie und gleichzeitig Chefarztnachfolger der Abteilung Neurologie des Klinikums Christophsbad in Göppingen. 1984 nahm er den Ruf auf die Professur für Neuroradiologie an der Universität Freiburg an und wurde Leiter der Sektion Neuroradiologie am Universitätsklinikum Freiburg. 2011 erfolgte offiziell seine Emeritierung. Äußere Umstände machten es erforderlich, dass er die neuroradiologische Verantwortung für weitere zwei Jahre übernahm, um dann eine technisch und personell exzellent ausgestattete neuroradiologische Klinik am 01.05.2013 an seinen Nachfolger Prof. Horst Urbach zu übergeben. Darüber hinaus blieb Prof. Schumacher bis zu seinem Tod der Neuroradiologie national und international eng verbunden.

Prof. Schumacher hat als Facharzt für Neurologie und Neuroradiologie stets Klinik, Diagnostik und Therapie als Einheit vertreten und gelehrt. Auch wenn er als einer der Pioniere der interventionellen Neuroradiologie in Deutschland unvergessen bleibt, so hat er immer an dieser Einheit und der engen Verbundenheit zu den klassischen Neurofächern Neurologie, Neurochirurgie und Psychiatrie festgehalten.

Auf seinen mehrmonatigen Forschungsaufenthalten mit neurointerventionellem Schwerpunkt (1984 in Paris bei Prof. Merland, 1986 in Pittsburgh bei Prof. Horton, bei Prof. Berenstein in New York und Prof. Kerber in San Diego sowie 1991 bei Prof. Viñuela in Los Angeles) stattete er sich mit dem notwendigen Rüstzeug aus, um innovative Behandlungen in Deutschland umzusetzen. Bereits 1986 begann er mit der regelmäßigen intraarteriellen Lyse beim akuten Schlaganfall und war einer der Ersten, der die mechanische Thrombektomie bereits Ende der 1990er-Jahre unterstützte und einführte. 1992 war er der erste Arzt in Deutschland und in Europa, der ein Hirnaneurysma mit elektrisch ablösbaren GDC-Coils behandelte.

Seine Begeisterung für die Neurointerventionen führte schon sehr früh zur Förderung der Forschungsaktivitäten in Freiburg. Bereits Anfang der 1990er-Jahre begann die systematische Ausbildung in Neurointerventionen in mehrtägigen Interventionskursen. Schumacher verstand es als Mensch, Lehrer und Wissenschaftler seine ärztlichen und nichtärztlichen Mitarbeiter zu begeistern und mitzunehmen.

Martin Schumacher war national und international für das Fach Neuroradiologie außerordentlich engagiert, er war für zwei Legislaturperioden Präsident der Deutschen Gesellschaft für Neuroradiologie (DGNR) sowie Präsident der Europäischen Gesellschaft für Neuroradiologie (ESNR), National Delegate der ESNR und der WIFTN sowie Vorsitzender der Arbeitsgemeinschaft für klinische Neurowissenschaften.

Neben der Ehrenmitgliedschaft der DGNR 2013 ehrten weitere nationale Gesellschaften Martin Schumacher für sein über die Landesgrenzen reichendes Engagement für die Neuroradiologie. Wir verdanken ihm, dass die UEMS die Subdivision Neuroradiology in der Section of Radiology gründete. Als Mitinitiator des *European Exchange Program* schuf er eine Möglichkeit, international noch enger zu kooperieren und auszubilden.

Für seine herausragenden Leistungen in der diagnostischen und interventionellen Neuroradiologie, aber auch als Persönlichkeit wurde er national und international sehr geschätzt.

Zu Martin Schumachers 60. Geburtstag hat sein Weggefährte Prof. Radü gesagt: *„Nur derjenige, der Grenzen überwindet, kann erfolgreich sein, und Martin Schumacher ist der Mann, der es verstand und versteht, geografische wie neuroradiologische Grenzen zu überwinden.“*

Viele enge Vertraute müssten an dieser Stelle genannt werden, die in tiefer Verbundenheit nun Abschied nehmen. Wir verbeugen uns und werden Martin Schumacher stets ein ehrendes Gedenken bewahren.

*Augsburg, München, Hannover im November 2021*.

*Prof. Dr. Ansgar Berlis, Prof. Dr. Claus Zimmer, Prof. Dr. Heinrich Lanfermann und der gesamte Vorstand der Deutschen Gesellschaft für Neuroradiologie e.* *V. und des Berufsverbandes Deutscher Neuroradiologen e.* *V. *

